# Recovery Mechanisms in Aged Kesterite Solar Cells

**DOI:** 10.1021/acsaem.1c03247

**Published:** 2022-03-08

**Authors:** Stephen Campbell, Martial Duchamp, Bethan Ford, Michael Jones, Linh Lan Nguyen, Matthew C. Naylor, Xinya Xu, Pietro Maiello, Guillaume Zoppi, Vincent Barrioz, Neil S. Beattie, Yongtao Qu

**Affiliations:** † Department of Mathematics, Physics and Electrical Engineering, Northumbria University, Newcastle upon Tyne NE1 8ST, United Kingdom; ‡ Laboratory for In Situ and Operando Electron Nanoscopy, School of Materials Science and Engineering, Nanyang Technological University, 637371 Singapore

**Keywords:** kesterite, photovoltaics, heat treatment, *in situ* transmission electron microscopy (TEM), elemental diffusion, SCAPS

## Abstract

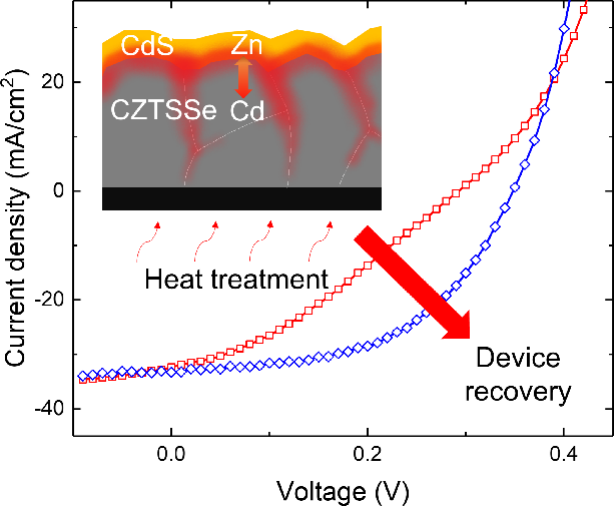

For successful long-term deployment and operation of kesterites Cu_2_ZnSn(S_*x*_Se_1–*x*_)_4_ (CZTSSe) as light-absorber materials for photovoltaics, device stability and recovery in kesterite solar cells are investigated. A low-temperature heat treatment is applied to overcome the poor charge extraction that developed in the natural aging process. It is suggested that defect states at aged CZTSSe/CdS heterojunctions were reduced, while apparent doping density in the CZTSSe absorber increased due to Cd/Zn interdiffusion at the heterojunction during the annealing process. *In situ* annealing experiments in a transmission electron microscope were used to investigate the elemental diffusion at the CZTSSe/CdS heterojunction. This study reveals the critical role of heat treatment to enhance the absorber/Mo back contact, improve the quality of the absorber/buffer heterojunction, and recover the device performance in aged kesterite thin-film solar cells.

## Introduction

Composed of Earth-abundant constituent elements, kesterites Cu_2_ZnSn(S_*x*_Se_1–*x*_)_4_ (CZTSSe) can be used as absorber materials in thin-film solar cells for indoor and outdoor applications including low-power-consuming electronic devices (few μW or W) in the Internet of Things (IoT) ecosystem.^1^ Kesterite also has the added benefit of solution-based fabrication via nanoparticle inks decoupling purification steps for better compatibility with high volume, ultralight substrates and boasts much lower-energy payback times.^2−5^ Intensive research has been conducted on CZTSSe in recent years. However, very few publications could be found focusing on aged CZTSSe solar cells to understand the device stability and recovery mechanisms for the long-term deployment and operation.

In this study, we noticed a poor charge extraction, and S-shaped current–voltage (*I*–*V*) characteristics were introduced in the natural aging process of CZTSSe solar cells under atmospheric conditions. As widely observed in the emerging solar cell materials and device architectures, S-shaped *I*–*V* curves (typically referred to as the S-kink) are detrimental to achieve high fill factors and power conversion efficiencies.^6−8^ Different solar cell material systems, including CdTe and CIGS, are found to have charge transport barriers, which limit the charge extraction at the selective contact interfaces.^6,9^ Here, we show the poor charge extraction and change in the material/electronic properties at the aged absorber/buffer interface can be overcome by annealing and doping via a low-temperature heat treatment, and the initial *I*–*V* characteristics can be recovered. The mitigation of the S-kink and device recovery mechanism in aged CZTSSe thin-film solar cells are investigated to provide physical insights and universal guidelines to remedy historically low open-circuit voltages (*V*_*OC*_) and conversion efficiencies of the next-generation kesterite technology.

The changes in CZTSSe solar cell performance and material properties during the recovery process were systematically studied using current density–voltage measurements under illumination (*J*–*V*) and in the dark at different temperatures (*J*–*V*–*T*) as well as by Raman plus photoluminescence (PL) spectroscopy, capacitance–voltage (*C*–*V*), and external quantum efficiency (EQE) measurement. *In situ* transmission electron microscopy (TEM) was used to investigate the elemental diffusion under annealing at the CZTSSe/CdS heterojunction in real time. Solar cell capacitance simulator (SCAPS) modeling is further used to understand the device recovery mechanism. We found that defect states at the CZTSSe/CdS interface were reduced in the heat treatment process, while the apparent doping density in the CZTSSe absorber increased due to Cd/Zn interdiffusion at the heterojunction. We also found the heat treatment enhanced the absorber/Mo back contact and contributed to the recovery of the device performance in aged kesterite thin-film solar cells.

## Results and Discussion

Kesterite solar cells used in this study were fabricated using CZTSSe absorbers from printable Cu_2_ZnSnS_4_ (CZTS) nanoparticle inks. CZTS nanoparticles were synthesized using a hot-injection method, and the resulting inks were deposited on molybdenum-coated sodalime glass (SLG) substrates via spin coating. A high-temperature step then followed to crystallize CZTS into CZTSSe absorbers. Solar cell devices were completed by addition of a CdS buffer layer (via chemical bath deposition) as well as window (intrinsic ZnO and conductive In_2_O_3_:SnO_2_ (ITO) by magnetron sputtering) and metal contact (Ni and Al by electron beam evaporation) layers. The fabrication process is described in greater detail in the Experimental Section and our previous works.^10−12^

**Figure 1:**
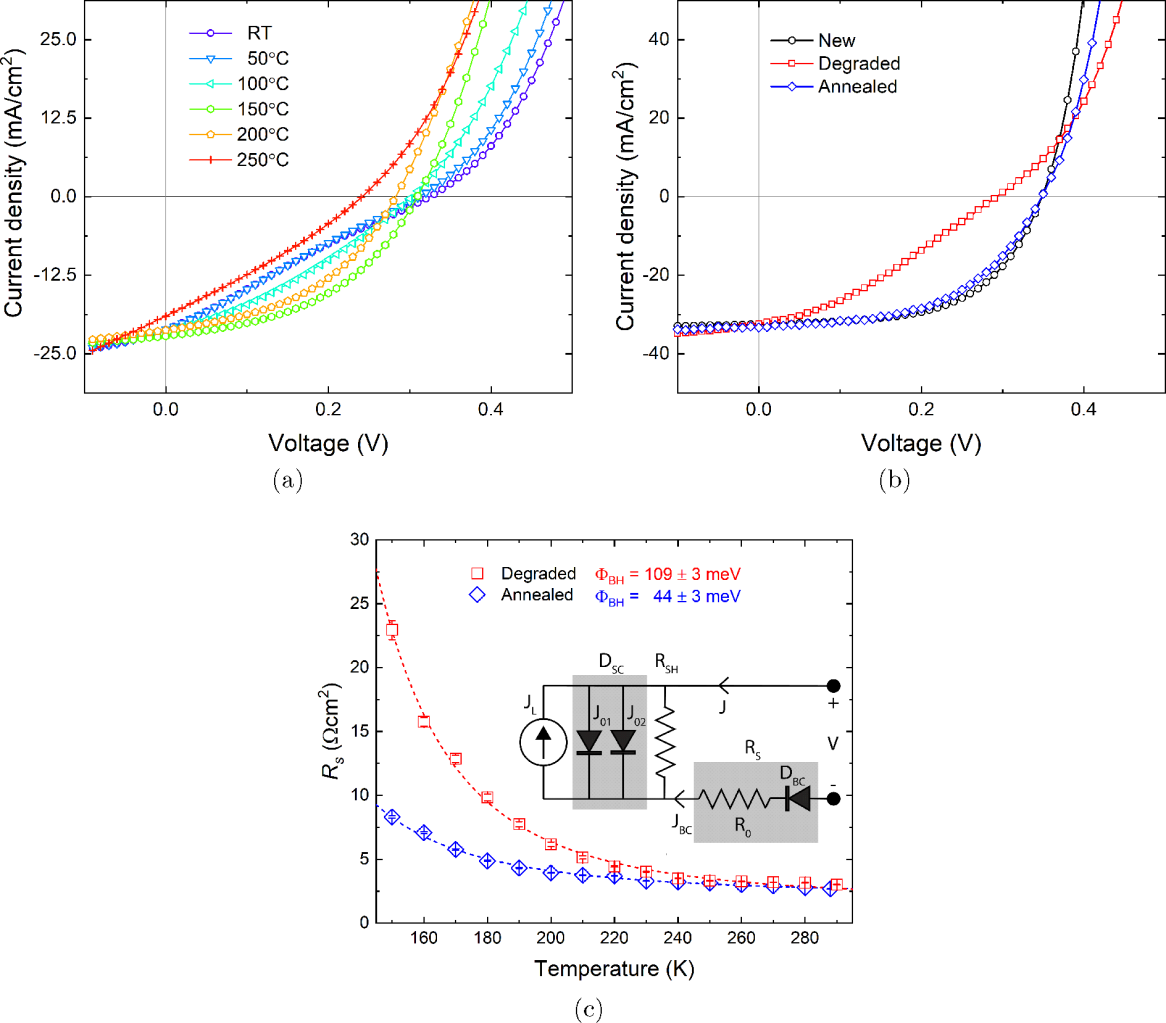
(a) *J*–*V* curves of a typical degraded CZTSSe device with different annealing temperatures together with (b) *J*–*V* curves of a newly fabricated, degraded (stored in ambient lab conditions for 10 months), and annealed (150 °C in Ar atmosphere for 10 min) CZTSSe solar cell and (c) series resistance as a function of temperature for the same degraded and annealed device where the dashed lines are a fit of eq 2. The inset shows the equivalent circuit model illustrating the solar cell diode, *D*_*SC*_ (comprising two recombination currents *J*_01_ in the QNR and *J*_02_ in the SCR), the photogenerated current source, *J*_*L*_, and the combined series resistance, *R*_*S*_ (consisting of background series resistance, *R*_0_, and a blocking back contact diode, *D*_*BC*_).

Previous studies have revealed postdeposition heat treatment (PDHT) of kesterite absorbers and solar cells leads to an improvement in all device parameters, such as *V*_*OC*_, short-circuit current density (*J*_*SC*_), fill factor (FF), and, correspondingly, efficiency (η).^13−17^ Here, we concentrate on the effect of PDHT on aged CZTSSe solar cells and demonstrate a recovery process in device performance. To determine optimal annealing conditions, a typical aged CZTSSe solar cell was heated from room temperature to 250 °C with *J*–*V* measurements obtained at regular intervals; see Figure 1a. The device was allowed to cool to room temperature following each annealing step before *J*–*V* measurements were performed. The *J*–*V* curve of the decayed solar cell shows an S-kink, which has greatly reduced the FF, *V*_*OC*_, and efficiency. After annealing, the FF of the degraded device improves significantly from room temperature to 150 °C before decreasing notably as the annealing temperature is increased further; see Figure S1. Consequently, the heat treatment recovery process was explored in detail using a temperature of 150 °C. The *J*–*V* characteristics of the solar cells studied were measured (i) when newly fabricated (subsequently referred to as “new” devices), (ii) after 10 months following storage under ambient lab conditions (room temperature, dark, 1 atm air—referred to as “degraded” devices), and (iii) immediately after a heat treatment of the degraded devices (150 °C in Ar atmosphere—referred to as “annealed” devices). When the degraded solar cells are annealed at 150 °C, it is interesting to find the initial *J*–*V* characteristics of the solar cell were mostly recovered, as shown in Figure 1b and Table 1. The increased series (*R*_*S*_) and decreased shunt (*R*_*SH*_) resistances observed in the degraded device significantly block the photogenerated current when the device is subjected to an increasing forward voltage bias. Once annealed, the series/shunt resistances return to values similar to a freshly fabricated device. This suggests a barrier to electron transport is present and could be mitigated with heat treatment. The increase in *R*_*S*_ observed in the degraded device in comparison to new and annealed devices can plausibly be explained by the presence of a Schottky potential energy barrier, such as a blocking back contact or a large “spike”-like positive conduction band offset at the buffer/absorber interface.^18^ The cause of the charge transport barriers and the degradation mechanism in kesterite thin-film solar cells are not detailed in this study; instead, we focus on the recovery process.

**Table 1:** *J*–*V* Device Parameters for the New, Degraded, and Annealed CZTSSe Device Obtained Using Double-Diode Analysis

	*V*_*OC*_	*J*_*SC*_	FF	η	*R*_*S*_	*R*_*SH*_	*J*_01_ (QNR)	*J*_02_ (SCR)
	(V)	(mA/cm^2^)	(%)	(%)	(Ω cm^2^)	(Ω cm^2^)	(mA/cm^2^)	(mA/cm^2^)
new	0.348	32.5	58.5	6.46	1.43	176	9.3 × 10^–5^	2.0 × 10^–1^
degraded	0.295	32.4	33.2	3.11	6.22	27.7	4.5 × 10^–5^	8.5 × 10^–1^
annealed	0.347	33.3	53.3	6.04	2.25	214	2.5 × 10^–6^	2.5 × 10^–1^

To investigate possible causes of the performance recovery, temperature-dependent dark *J*–*V* measurements (*J*–*V*–*T*) were carried out when the solar cell was degraded and subsequently annealed. *R*_*S*_ can be extracted from the following relationship
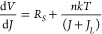
1where *n*, *k*, *T*, *q*, and *J*_*L*_ are the diode ideality factor, Boltzmann constant, temperature, electron charge, and photogenerated current density, respectively (see Figure 1c).^20^ The blocking back contact issue is evident in the diverging *R*_*S*_ with decreasing temperature for both degraded and annealed cells.

Gunawan et al. proposed a model to describe the temperature-dependent behavior of the series resistance, where
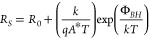
2Here, A* is the effective Richardson constant, Φ_*BH*_ is the barrier height, and *R*_0_ is the background series resistance, due to the front contact and bulk resistance, which is usually small and nominally temperature-independent and therefore neglected in this analysis.^19^ The exponential (Schottky) term relates to the thermionic emission model, arising from the blocking back contact. In this model, the solar cell equivalent circuit comprises the main solar cell (with diodes representing the space charge region (SCR) and quasineutral region (QNR) collectively referred to as *D*_*SC*_) and back contact diode *D*_*BC*_; see the inset of Figure 1c. When the solar cell is in a forward-biased condition, the back contact diode is in reverse bias. In this state, the reverse saturation current of the back contact diode (*J*_*BC*_) limits its conduction. As the device temperature is lowered toward cryogenic temperatures, the reverse saturation current diminishes rapidly, and *R*_*S*_ increases as a consequence.

From fitting of eq 2, blocking contact barrier heights of 109 ± 3 and 44 ± 3 meV were evaluated for the degraded and annealed devices, respectively. The barrier height observed in the annealed device is in good agreement with that determined in our previous study.^21^*J*–*V* curves in Figure 1b were further fitted using the double-diode model for a n+–p device under illumination (see Figure S2), described by
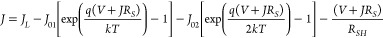
3in order to extract reverse saturation currents *J*_01_ and *J*_02_ to understand the recombination currents related to the QNR and SCR of the solar cell, respectively. It is apparent the degraded device is limited by *J*_02_, which increases as the device ages, rising from 0.20 to 0.85 mA/cm^2^ after 10 months. However, heat-treating the device leads to a recovery of *J*_02_ (0.25 mA/cm^2^), suggesting there is some change in the material/electronic properties of the absorber in the SCR. The extracted Φ_*BH*_ values are entirely consistent with the observed lower *J*_01_ and *J*_02_ values for the annealed cell, where the *J*_*BC*_ of the back contact diode in this device would also be expected to be lower. This result suggests that the absorber/Mo back contact is recovered following the heat treatment, which agrees with our previous study on newly fabricated CZTSSe devices.^21^ The underlying reason for the reduction in Φ_*BH*_ upon annealing the degraded device is unclear but is probably not related to the MoSe_2_ layer at the Mo back contact, where the layer thickness was unchanged after the heat treatment (see Figure S3).

**Figure 2:**
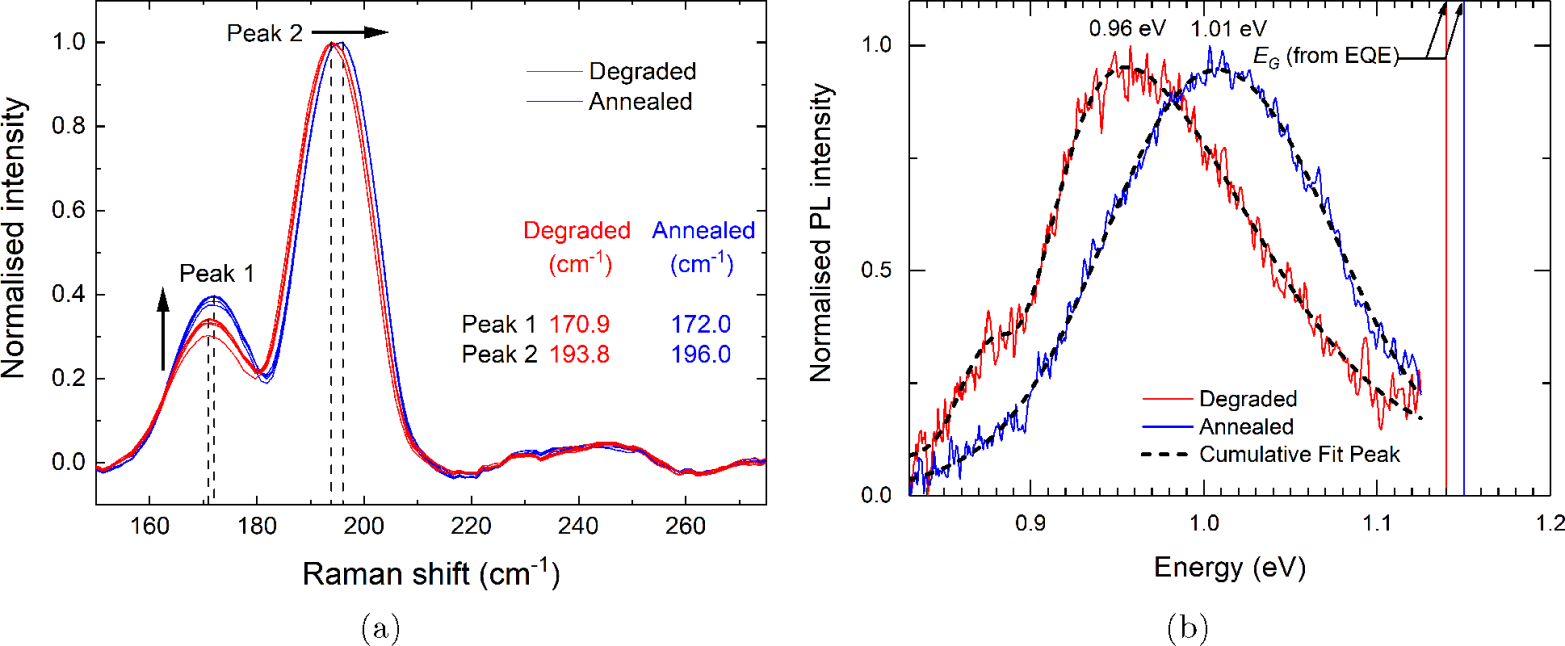
(a) Normalized Raman spectra from multiple measurements of the degraded and annealed CZTSSe device. A slight blue shift of the two most intense peaks as well as an increase in the relative intensity of the 172 cm^–1^ (B mode) peak is observed after the degraded device is annealed and (b) normalized room temperature PL spectra of the degraded and annealed CZTSSe device with corresponding CZTSSe absorber bandgaps *E*_*G*_ determined from external quantum efficiency (EQE) measurements.

Raman measurements were performed on a representative degraded device and on the same device immediately after 150 °C annealing, with normalized results presented in Figure 2a. It is possible to probe the CZTSSe absorber layer of the complete solar cell , as the *i*-ZnO, ITO, and CdS layers are almost transparent to the 633 nm laser used here. Raman measurements mainly probe the top region of the CZTSSe absorber close to the heterojunction as the penetration depth of 633 nm laser wavelength is around 250 nm.^22^ As shown in Figure 2a, the two sharp peaks at 172 (peak 1) and 195 cm^–1^ (peak 2) and a weak peak at 235 cm^–1^ (peak 3) are consistent with the B, A, and E modes of CZTSe, respectively.^23−25^ A multiple-Gaussian fit (see Figure S4a) was applied to the Raman spectra to elicit peak height and position.

As seen in Figures 2a and S4b, the intensity of the B mode peak at ∼172 cm^–1^ increases for the device following heat treatment, and the fwhm decreases slightly (from an average of 14.2 degraded to 13.5 cm^–1^ annealed). This behavior has been observed previously and was associated with a decrease in the density of the defect cluster [V_Cu_ + Zn_Cu_].^26^ Rey et al. also found that the decreasing/broadening of the 170 cm^–1^ peak (observed in our degraded cell) was indicative of an order–disorder transition of CZTSe, i.e., an increase in the concentration of Cu_Zn_ and Zn_Cu_ antisite defects produces a more disordered absorber. Another study noted the presence of V_Cu_ and Zn_Cu_ point defects in CZTSe causes a decrease in the B symmetry mode peak around 170 cm^–1^ and is related to vibrations in the Cu/Zn and Cu/Sn atomic planes (meaning a decrease in the Cu/Zn and Cu/Sn vibrational units).^27^ As the opposite effect is seen upon annealing the degraded device, it can be assumed that there is a decrease in the density of these antisite defects. Also, Neuschitzer et al. states that the intensity increase in the B mode peak is related to Cu enrichment of the surface region of the CZTSe absorber and an increase in Cu/Zn ordering.^13^ It was also found that an increase in Cu/Zn ordering in CZTSe moves the main Raman peaks toward higher Raman shifts.^26^ In addition to the increase in the B mode peak in the annealed device, both A and B mode peaks are shifted to higher values, specfically from the A mode at 193.8 cm^–1^ and B mode at 170.9 cm^–1^ in the degraded device to the A mode at 196.0 cm^–1^ and B mode at 172.0 cm^–1^ in the annealed device. This is further evidence that heat-treating the degraded device induces changes in the material properties of the buffer/absorber interface and near-surface region of the CZTSSe film. Hence, the absorber quality at heterojunction is improved following the annealing process.

Room temperature photoluminescence (PL) spectra plotted in Figure 2b show one main broad peak centered at 0.96 and 1.01 eV together with a smaller peak at 0.87 and 0.95 eV for degraded and annealed devices, respectively. Each PL spectrum was fitted with two asymmetric double-sigmoidal functions (see Figure S5). For the degraded device, the lower-energy value of the main PL peak maximum compared to value of the corresponding absorber bandgap *E*_*G*_ (measured by external quantum efficiency (EQE) discussed in detail below) has been linked to an increase in band tailing and cation Cu/Zn disorder.^17,26^

**Figure 3:**
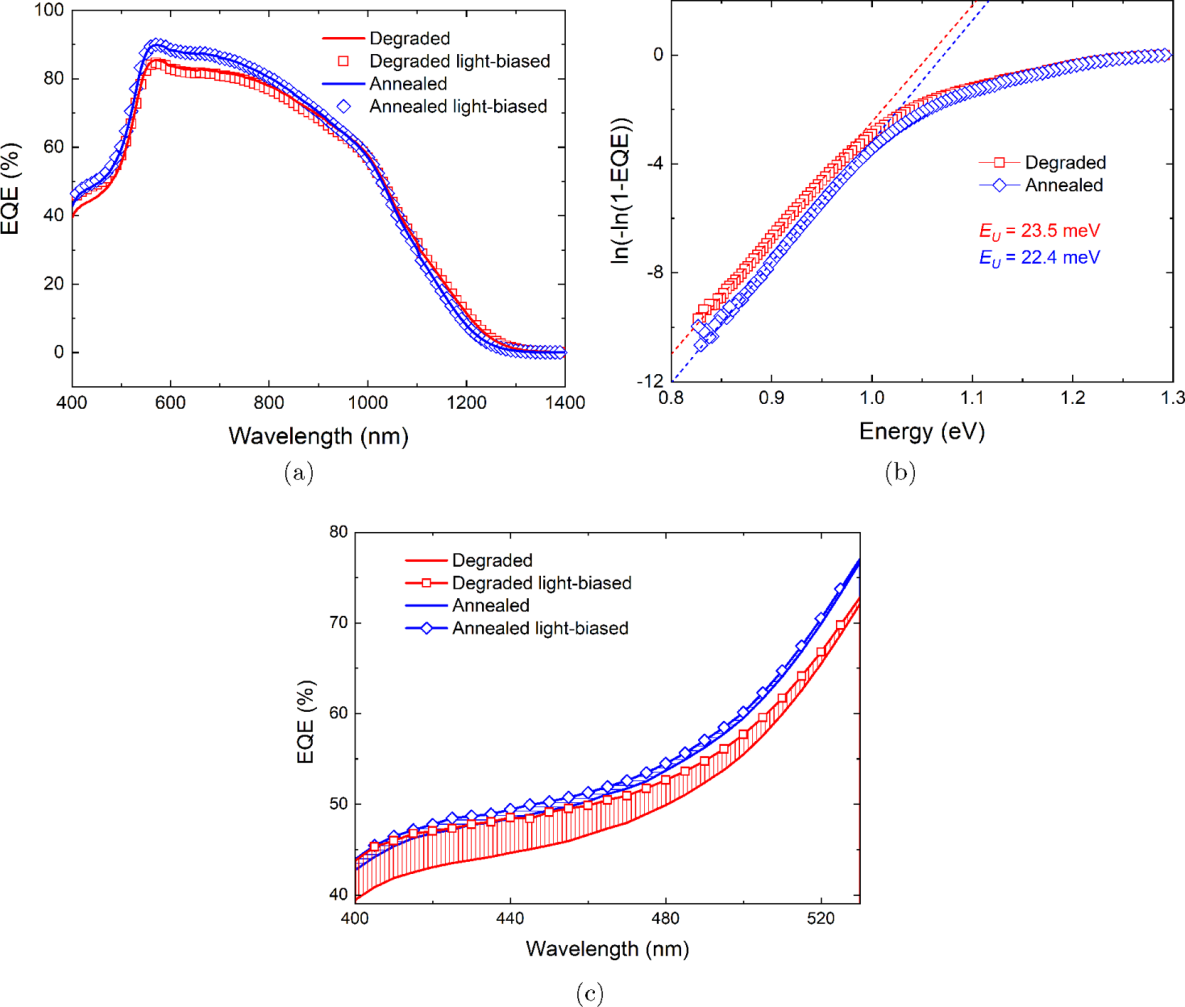
(a) EQE spectra of solar cells before and after heat treatment with light-biased measurements under 1.55 mW/cm^2^ illumination, (b) the Urbach tail energy, *E*_*U*_, extracted from the unbiased EQE data, and (c) enlarged dark and light-biased EQE plots in the low-wavelength range.

Figure 3a shows EQE spectra of the degraded and annealed CZTSSe solar cell under normal and white-light-biased conditions. *J*_*SC*_ obtained from integration of the area under the unbiased EQE plots gives values of 29.8 and 30.7 mA/cm^2^ for the degraded and annealed device, respectively. A similar difference of around 1 mA/cm^2^ in *J*_*SC*_ is observed for the degraded/annealed device when *J*_*SC*_ is extracted from *J*–*V* plots (see Figure 1b and Table 1). Additionally, the Urbach tail energy (*E*_*U*_) can be extracted from the unbiased EQE data; see Figure 3b. *E*_*U*_ can be determined from the slope of the linear region below the respective bandgaps of the degraded and annealed absorbers (i.e., the slope is equal to 1/*E*_*U*_) and gives *E*_*U*_ values of 23.5 and 22.4 meV for the degraded and annealed devices, respectively.^28^ A well-known phenomenon in CZTSSe is band tailing related to a high density of Cu–Zn antisite defect pairs, also known as Cu–Zn disorder.^29^ Therefore, the reduction in *E*_*U*_ observed for the annealed device suggests the Cu–Zn disorder is reduced upon annealing the device, which is consistent with the Raman and PL results. EQE measurements with white light bias (1.55 mW/cm^2^) were also performed. As highlighted in Figure 3c, the degraded device shows a higher carrier collection in the low-wavelength range (400–500 nm) under light bias, while no obvious difference is observed once the device is annealed. This could be related to the suppression of light-activated defects in the CdS layer of the annealed device.^30^ The degraded device shows recombination centers forming in the CdS layer, which have been rectified by annealing, and greater absorption is seen in the whole device. The bandgap energy (*E*_*G*_) of the CZTSSe film was estimated from the EQE data by extrapolating the [hυ*ln(1 – EQE)]^2^ vs *hυ* plot to the photon energy, as shown in Figure S6. A small increase in the bandgap is seen when the device was annealed, from 1.14 eV when degraded to 1.15 eV, suggesting there is a slight compositional change or greater degree of ordering in the absorber. We speculate that the reduction in band tailing in the annealed device is related to a decrease in Cu/Zn disorder with the moderation of [V_Cu_ + Zn_Cu_] defect clusters. Combined with previous Raman and PL analysis, we can see that annealing not only improves the quality of the CZTSSe/buffer heterojunction but also modifies the material/electronic properties of the near-interface region of the CZTSSe absorber.

**Figure 4:**
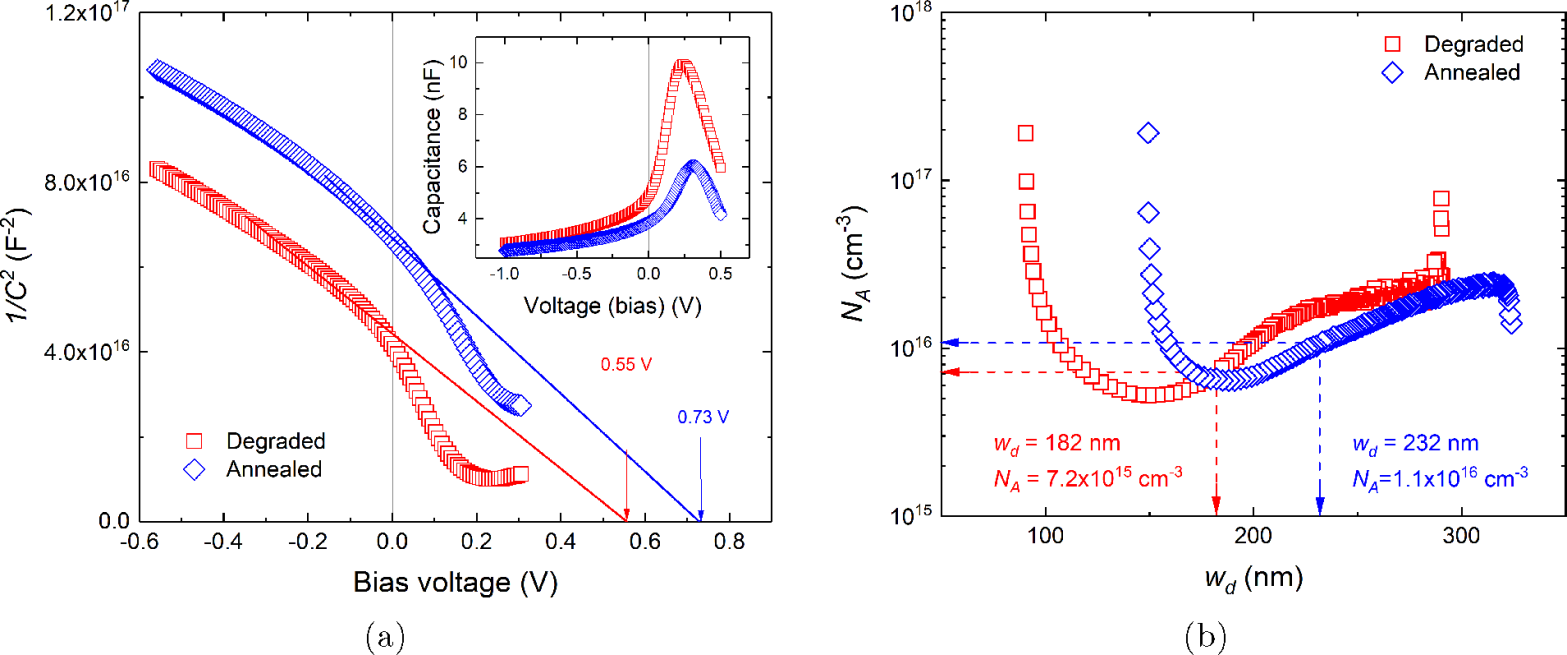
(a) Mott–Schottky plots of the degraded and annealed CZTSSe device with a linear fit of the reverse bias region indicating the built-in voltage *V*_*bi*_ of each device. The inset shows the capacitance–voltage (*C*–*V*) plots of corresponding solar cells and (b) the carrier concentration depth profiles with indicated *w*_*d*_ and *N*_*A*_ values at zero bias.

Capacitance–voltage (*C*–*V*) measurements (inset of Figure 4a) were conducted on the degraded and annealed device to further investigate the performance variations. A Mott–Schottky (MS) plot (Figure 4a) is made from the *C*–*V* data using the following relationship
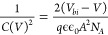
4where *q*, ϵ_0_, ϵ, *A*, *N*_*A*_, and *V*_*bi*_ are the electron charge, vacuum permittivity, dielectric constant of CZTSSe, cell area, carrier density, and built-in voltage, respectively. The *V*_*bi*_ of each device is determined by extrapolating the linear region of the reverse voltage bias in the MS plot and taking the intercept of the voltage axis. The linear fit is typically done using the reverse voltage range, as this region is where the capacitance is dominated by the pseudocapacitor of the depletion layer (see inset of Figure 4a).^31^*V*_*bi*_ values of 0.55 and 0.73 V were ascertained for the degraded and annealed devices, respectively. An increase in *V*_*bi*_ is correlated to a higher *V*_*OC*_ in the annealed device, where *V*_*OC*_ rises from 0.295 V in the degraded device to 0.347 V following heat treatment. The increase in *V*_*bi*_ can be explained by an overall increase in the depletion region width (*w*_*d*_). *w*_*d*_ can be expressed as a function of capacitance according to
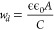
5

A plot of *N*_*A*_ vs *w*_*d*_ is produced to quantify the change annealing treatment has on the depletion width and carrier concentration across the profile of the absorber, shown in Figure 4b. An increase is seen in both *w*_*d*_ and *N*_*A*_ with annealing treatment. The increase in the depletion region width from 182 nm (degraded) to 232 nm (annealed) can be attributed to a change in the SCR, assumed to be a result of an improvement of the CZTSSe/CdS interface and is entirely consistent with Raman, PL, and EQE analysis. The carrier concentration of the device increases from 7.2 × 10^15^ to 1.1 × 10^16^ cm^–3^ following the 150 °C annealing. A number of investigations have highlighted the beneficial effects of PDHT on kesterite/buffer heterojunction films and complete solar cells, citing the interdiffusion of Cd and Zn elements across the CdS/CZTSSe interface as the main cause of increased carrier density and improvements in material/device properties.^13−17^

**Figure 5:**
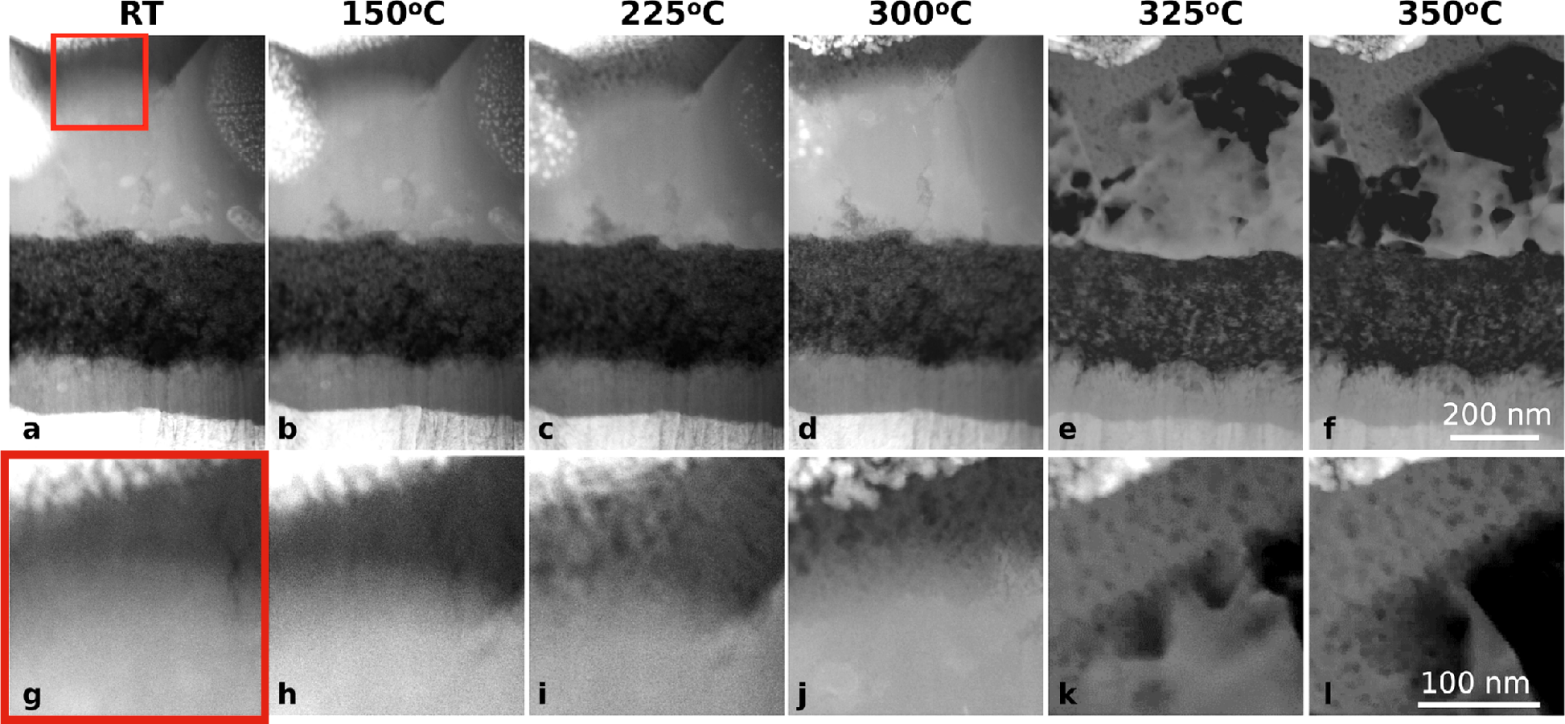
(a–f) Low magnification and (g–l) higher magnification of a cross-sectional HAADF-STEM image of degraded solar cell lamellae annealed *in situ* in a TEM recorded from room temperature to 350 °C. The higher-magnification images are from the CdS/CZTSSe interface where the degradation of both layers is visible in both layers with increasing temperature, albeit starting at lower temperature for the CdS layer.

**Figure 6:**
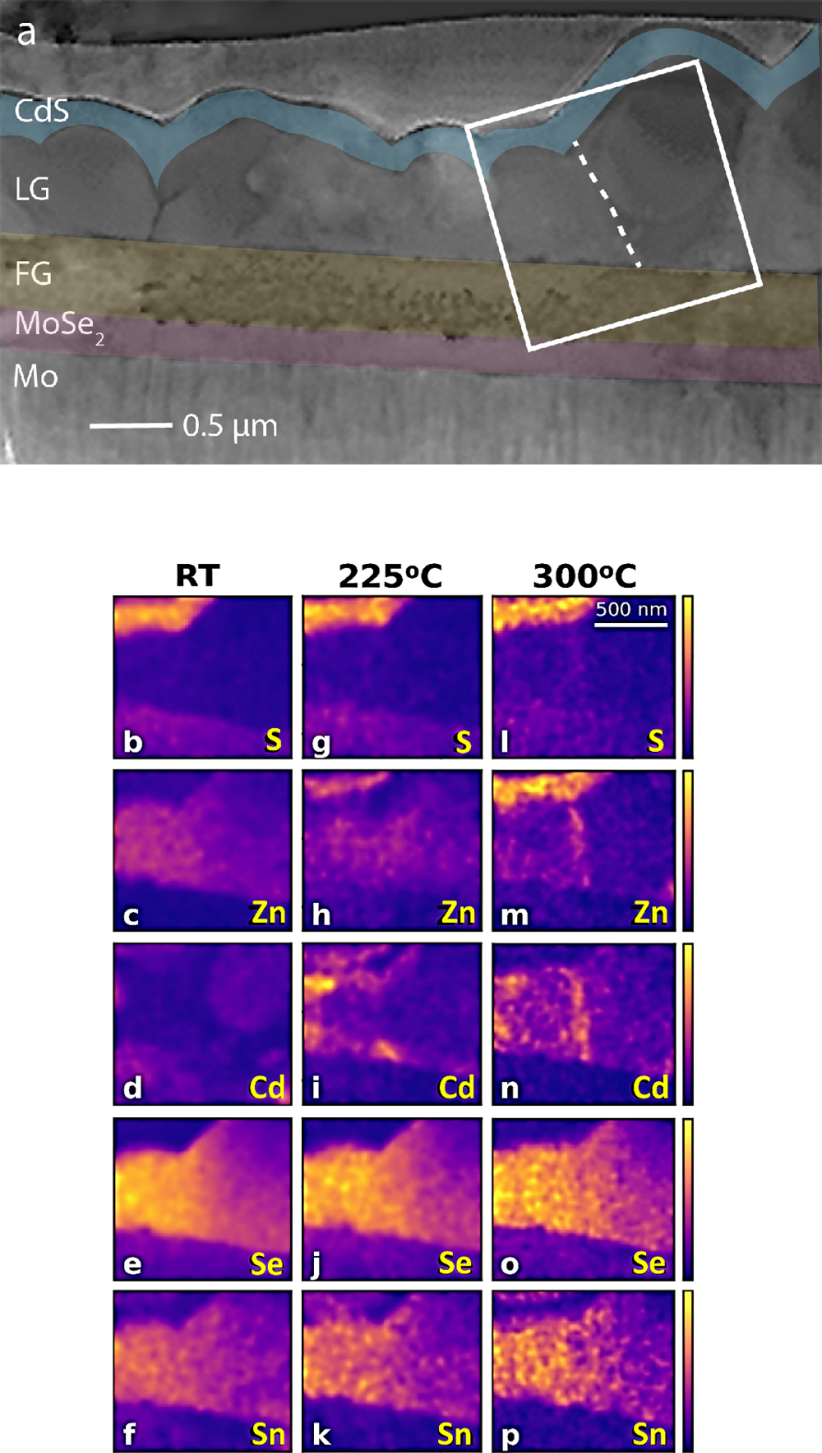
(a) Cross-sectional HAADF-STEM image of a CZTSSe/CdS heterojunction lamellae annealed *in situ*, displayed in false colors. The component layers, including CdS, the large-grain (LG) absorber layer, the fine-grain (FG) absorber layer, MoSe_2_, and the Mo layer are listed from top to bottom. A dashed line is used to highlight a grain boundary in the LG layer. A top platinum (Pt) layer is coated for the specimen preparation. (b–p) Normalized elemental EDX mappings of the white line enclosed area shown in (a) recorded at (b–f) RT as well as after annealing at (g–k) 225 °C and (l–p) 300 °C. Each row is made of three STEM–EDX maps corresponding to a given element, starting from the top (b,g,l) S, (c,h,m) Zn, (d,i,n) Cd, (e,j,o) Se, and (f,k,p) Sn.

The microstructure and elemental diffusion across the CZTSSe and CdS buffer interface under thermal annealing were investigated *in situ* using TEM. Figure 5 displays the cross-sectional scanning (S)TEM images of a CZTSSe layer with a thin film of ∼100 nm CdS deposited on top to form the heterojunction annealing from room temperature (RT) to 350 °C. A HAADF-STEM image with a larger field of view is shown in Figure 6a, and it reveals the CZTSSe solar cell multilayered structure. The large-grain (LG) layer is composed of high-purity CZTSSe with low carbon content. The residual fine-grain (FG) layer is rich in carbon from the long hydrocarbon chains of the ink solvents. The bottom substrate layer is the columnar grain structured Mo with a MoSe_2_ layer on top formed under the selenization conditions. More discussion on the origin of the four compositional layers is published elsewhere.^21^

In order to study the interdiffusion between the different layers, *in situ* annealing of the full solar cell stack was performed inside the TEM. The changes in the microstructure and in composition were followed by HAADF-STEM and STEM–EDX imaging. The HAADF-STEM imaging technique is sensitive to compositional variations and thus is ideal to study the interdiffusion at the interfaces. Figure 5a–f shows the evolution of the full stack of the solar cell microstructure for increasing temperatures. In these low-magnification images, the CZTSSe layer decomposes at 325 °C, while it seems to be stable for temperatures of 300 °C and below. A closer look at the CdS/CZTSSe interface, shown in Figure 5g–l, shows the presence of voids in the CdS layer for annealing temperatures of 150 °C and above. More pronounced changes are visible in the CdS layer for temperatures of 225 °C and above. This confirms the hypothesis established above from electrical and optical characterisations that a modification of the local composition at the CdS/CZTSSe is taking place upon annealing at 150 °C. Figure 6b–p shows the STEM–EDX maps of the initial solar cell layers at RT and after annealing at 225 and 300 °C. At these temperatures, the integrity of the CZTSSe layer is preserved, but voids are clearly formed in the CdS. The STEM–EDX imaging mode is less sensitive to small variations in concentrations but allows identification of the elemental species that diffuse during the annealing process. The diffusion process upon annealing was followed by comparing the STEM–EDX maps recorded at RT with maps recorded at 225 and 300 °C, for which noticeable differences are visible.

The STEM–EDX maps of all the elements in the CZTSSe and CdS layers are shown in Figure 6, except for Cu, as unwanted X-ray contributions from the TEM column led to nonexploitable Cu signal. The distribution of Se, Sn, Zn, and S is what we expect for homogeneous CZTSSe and CdS layers. The contrast variation within the layers is due to thickness variation, resulting in lower X-ray signals for thinner areas. The nonuniform distribution of the Cd maps at RT is likely due to the FIB milling process used to prepare the TEM lamella (see the Experimental Section for more details). For the specimen annealed to 225 °C, a clear elemental interdiffusion is observed in the heterojunction region as shown in Figure 6g–k. On the buffer side, Zn was found to have diffused into the CdS layer. On the CZTSSe layer, a significant amount of Cd had diffused at the contact points between the grain boundaries and the FG and CdS layers upon annealing. As the temperature reaches 300 °C, the Cd is redistributed through the full length of the grain boundaries within the absorber layer. The Zn diffused both upward into the CdS layer and at the grain boundaries within the absorber layer. In addition to Cd/Zn diffusion, there is no obvious redistribution of the other elements at elevated temperatures. The Cd/Zn diffusion between the heterojunction is well-demonstrated for extreme conditions at 225 and 300 °C. Despite these temperatures being larger than the one used for the electrical characterization, we can observe the modification of the CdS layer is already visible for 150 °C annealing in the *in situ* TEM. The higher temperatures used in the *in situ* annealing TEM experiments allowed for an increase in the diffusivity of the mobile elements, making the Cd/Zn migration within the studied solar cell device more observable.

It is well-established that a Zn-alloyed CdS buffer allows a better response in the short-wavelength region and a favorite “spike”-like band alignment.^32^ It is also true that Cd-alloyed kesterite solar cells demonstrate high-efficiency potential by reducing the *V*_*OC*_ deficit and recombination issue.^33^ However, an excessive interdiffusion could be detrimental as shown in Figures 1a and S1 where device performance deteriorated notably once the annealing temperature exceeded 200 °C. Elemental distribution at lower temperatures and fine-tuning of Cd/Zn interdiffusion across the heterojunction requires further investigation.

**Figure 7:**
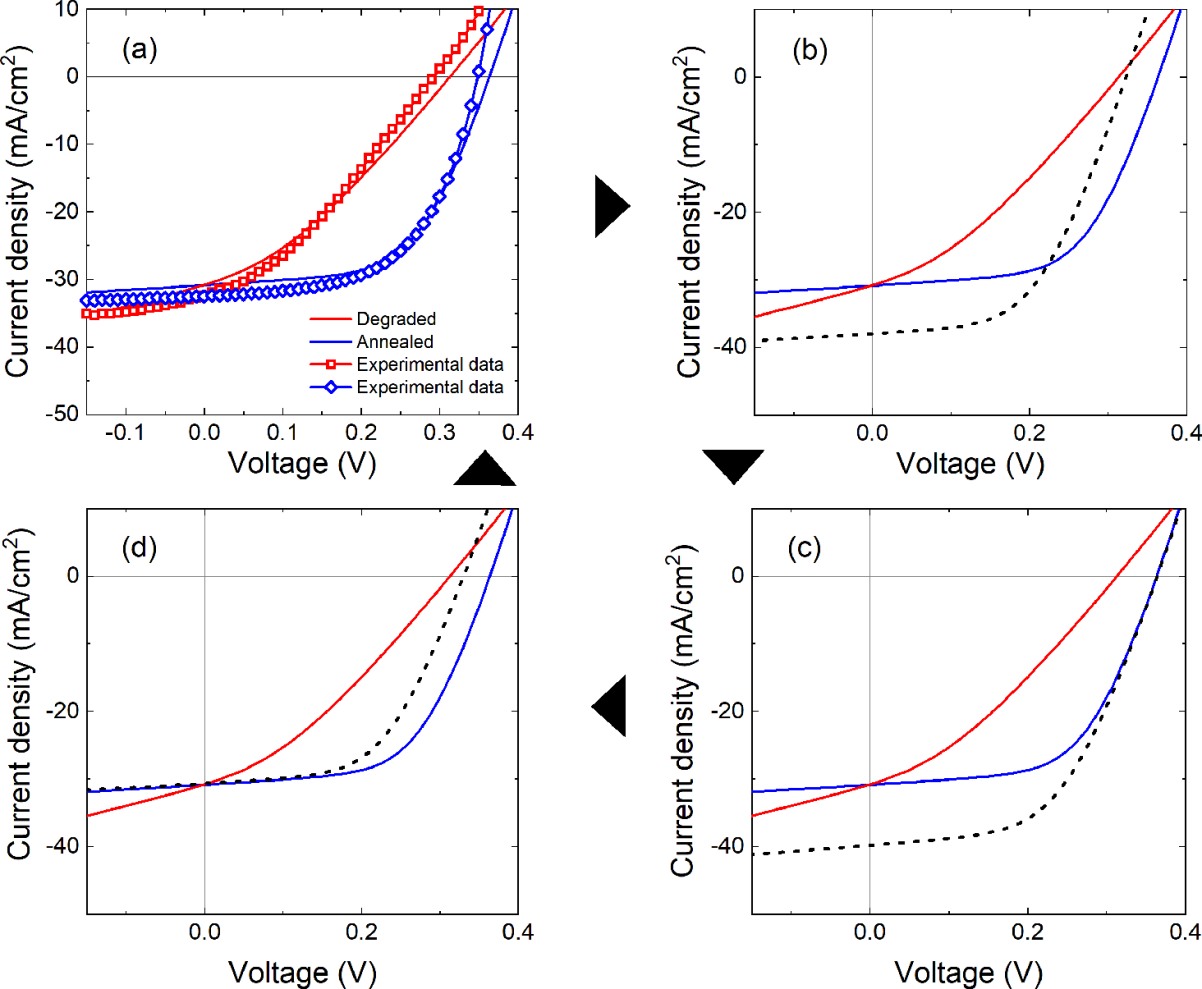
*J*–*V* curves of SCAPS simulations for a typical degraded and annealed CZTSSe solar cell. Clockwise from (a) are simulated *J*–*V* curves that closely match the experimental plots in Figure 1b. Experimentally determined device parameters (*R*_*S*_, *R*_*SH*_, *N*_*A*_, *E*_*G*_, and Φ_*BH*_) for the degraded device were changed to the values determined for the annealed device, with the corresponding *J*–*V* plot shown as a dashed black line in (b).The *J*_*SC*_ is overestimated and *V*_*OC*_ is underestimated compared to the simulated annealed device. In (c), the density of the heterojunction interface defects (*N*_*int*_) were then reduced in the degraded device in order to improve *V*_*OC*_. At a specific value of *N*_*int*_, the *V*_*OC*_ matched the simulated annealed device, but *J*_*SC*_ remained excessive. Next, the concentration of a deep acceptor defect (*N*_*acc*_) in the CZTSSe bulk near the CdS/CZTSSe interface was increased to lower the *J*_*SC*_ of the degraded device with the result shown in (d). Now, the *J*_*SC*_ matches that of the simulated annealed device, but the *V*_*OC*_ has dropped below the annealed device value. The interface defect density in the degraded device was further reduced until the *J*–*V* curve replicated that of the annealed device, as shown in (a). The optimized values of *N*_*int*_ and *N*_*acc*_ are shown in Table 2.

To gain an insight into the physical processes taking place when a degraded CZTSSe solar cell is heat-treated, device simulations using SCAPS were performed such that material properties of the simulated degraded cell were optimized to replicate a simulated annealed device. Experimentally determined *J*–*V*, *C*–*V*, and EQE parameters of the degraded and annealed solar cell were used in the simulations, and a summary of all material properties are listed in Table S1. Figure 7a shows the simulated *J*–*V* curves for the degraded/annealed device and are a very good approximation to those of the actual device (see Figure 7a). The simulated device parameters are listed in Table 2.

**Table 2:** Extracted *J*–*V* Parameters from SCAPS Simulations of a Degraded and Annealed CZTSSe Solar Cell Together with CdS/CZTSSe Interface (*N*_*int*_) and Bulk Acceptor (*N*_*acc*_) Defect Densities

	*V*_*OC*_	*J*_*SC*_	FF	η	*R*_*S*_	*R*_*SH*_	*N*_*int*_	*N*_*acc*_
	(V)	(mA/cm^2^)	(%)	(%)	(Ω cm^2^)	(Ω cm^2^)	(cm^–3^)	(cm^–3^)
degraded	0.313	30.8	32.6	3.14	6.22	27.7	2.3 × 10^14^	2.0 × 10^16^
annealed	0.364	30.9	55.6	6.16	2.25	214	2.2 × 10^13^	4.5 ×10^18^

In order to accurately replicate the real *J*–*V* curves of the degraded/annealed device, two additional parameters were introduced to the simulations, specifically the buffer/absorber interface defect density (*N*_*int*_) and acceptor-type defect density in the absorber (*N*_*acc*_). Due to the interdiffusion of Cd/Zn promoted by heat treatment across the heterojunction, it is reasonable to expect the formation of Cd-based antisite defects within the region of the CZTSSe absorber.^14^ First-principles studies show the formation energy of Cd_Zn_ is lower than that of Cd_Cu_ and Cd_Sn_, suggesting this is the prevalent Cd-related acceptor defect in the upper region of the CZTSSe film.^33^ The diffusion of Cd into the absorber, i.e., the diffusion gradient of acceptor defect Cd_Zn_ from the absorber/buffer interface, was modeled in SCAPS using diffusion: complementary error function law.^34^

Initially, only the experimentally determined parameters (*R*_*S*_, *R*_*SH*_, *N*_*A*_, *E*_*G*_, and Φ_*BH*_) were changed for the degraded device with *J*–*V* results shown as a dashed black curve in Figure 7b. The simulation overestimates *J*_*SC*_ and underestimates *V*_*OC*_ compared to the curve for the annealed device. Next, the concentration of interface defects (*N*_*int*_) was reduced from the degraded device value of 2.4 × 10^14^ to 5.2 × 10^13^ cm^–3^ in order to boost *V*_*OC*_. The simulated curve in Figure 7c shows that the *V*_*OC*_ matches that of the annealed device, but *J*_*SC*_ is still excessive. From the previous discussion, it is reasonable to expect an increase in the Cd_Zn_ antisite defect concentration in the CZTSSe absorber following an annealing step. The density of this defect (*N*_*acc*_) was subsequently increased from the degraded device value of 2.0 × 10^16^ to 4.5 × 10^18^ cm^–3^ with results shown in Figure 7d. The *V*_*OC*_ of the simulated curve drops as a result of the increase in Cd_Zn_ defect concentration. Finally, the interface defect density was readjusted from 5.2 × 10^14^ down to 2.2 × 10^13^ cm^–3^, and the simulation data now accurately match the *J*–*V* curve of the simulated annealed device as seen in Figure 7a. Thus, simulation results have demonstrated the recovery processes in degraded device performance upon a 150 °C annealing, which could relate to (i) a reduction of absorber/buffer interface defect states and (ii) elemental Cd/Zn interdiffusion at the CdS/CZTSSe heterojunction where Cd diffusion into the SCR of the CZTSSe absorber forms acceptor-type Cd_Zn_ defects, increasing the apparent doping density of absorber layer.

In summary, poor charge extraction and an S-kink were introduced in aged CZTSSe solar cells. Upon application of a low-temperature heat treatment of 150 °C to degraded solar cells, initial *J*–*V* characteristics are recovered due to an enhancement of the absorber/Mo back contact and improvement in the quality of the absorber/buffer heterojunction. By performing *in situ* annealing inside a TEM, Cd/Zn interdiffusion accross the CZTSSe/CdS interface was directly observed during the heat treatment. This insight obtained in real time and validated by SCAPS device simulations reveal the S-kink mitigation mechanism. This study demonstrates the importance of a quality absorber/buffer interface in achieving high *V*_*OC*_ and efficient solar cells and explains the positive effects of elemental diffusion, pointing a way forward for device recovery in aged thin-film solar cells.

## Experimental Section

### CZTS Nanoparticle Inks

CZTS nanoparticles were fabricated using a hot-injection method where a sulfur–oleylamine (OLA) solution was injected into a hot metallic precursor–OLA solution under air-free conditions. The following metallic precursor molar ratios were chosen: Cu/(Zn + Sn) = 0.79 and Zn/Sn = 1.27, achieved by using 1.34 mmol of copper acetylacetonates (Cu(acac)_2_), 0.95 mmol of Zn(acac)_2_, and 0.75 mmol of Sn(acac)_2_Cl_2_ as the metallic source to guarantee a Cu-poor, Zn-rich composition region for high solar cell efficiencies. After the reaction at 225 °C for 30 min, the as-synthesized nanoparticles were precipitated and washed twice by using isopropanol (IPA) and toluene. The collected CZTS nanoparticles were dispersed in 1-hexanethiol with the aid of sonication to provide CZTS nanoparticle inks with a concentration of ∼100 mg/mL.

### Kesterite Absorber Fabrication

CZTS precursor thin films were deposited by spin coating. CZTS nanoparticle inks were applied to onto molybdenum-coated SLG substrates at a speed of 1200 rpm for 5 s. The samples were then dried on a hot plate at 150 °C for 30 s and then at 300 °C in air for 30 s (“soft-baking”). A thickness of ∼1 μm was deposited by repeating spin-coating and soft-baking procedures. The as-deposited CZTS thin films were then annealed with ∼300 mg of selenium pellets in a tube furnace. The furnace was evacuated (6.0 × 10^–3^ mbar), and a backing Ar atmosphere (∼150 mbar) was provided before the temperature was raised to 500 °C and held constant for 20 min.

### Solar Cell Fabrication

The absorber films were integrated into solar cell devices following the deposition of CdS, intrinsic ZnO (i-ZnO), ITO, and Ni/Al contact layers. The CdS buffer layer was ∼70 nm thick and deposited by a chemical bath process using cadmium sulfate as the cadmium source, thiourea as the sulfur source, and ammonium hydroxide to adjust pH to ∼11. The transparent oxide layers were deposited by magnetron sputtering using ∼60 nm of i-ZnO and ∼200 nm of ITO. Finally, the front contact grid was deposited by electron beam evaporation of Ni (∼50 nm) and Al (∼1 μm) through a shadow mask. The total area of final solar cells was ∼0.16 cm^2^ defined by mechanical scribing.

### Solar Cell Heat Treatment

The degraded solar cells were loaded into a tube furnace on an aluminum foil tray. The tube furnace was first flushed with Ar for 5 min before the temperature was increased from room temperature to 150 °C in 15 min. The temperature was then held at 150 °C for 10 min to anneal the solar cells under a slow Ar flow (0.3 L/min). After the annealing treatment, the samples were extracted from the heating zone of the tube furnace and cooled down rapidly under a fast Ar flow (2 L/min). Once the temperature was lower than 100 °C, Ar flow was stopped, and the solar cells were left in the tube furnace to cool down naturally to room temperature overnight, before the *J*–*V* measurements were repeated.

### Solar Cell Measurement

The current–voltage characteristics of solar cells were measured in a four-point probe configuration using a Keithley 2400 series source meter. Solar cells were illuminated with an Abet Technologies Sun 2000 solar simulator with an air mass 1.5 spectrum set at 100 mW/cm^2^. *J*–*V*–*T* measurements were performed to measure the dark *J*–*V* curves of solar cells in a cryostat at each temperature from 290 to 150 K in 10 K increments. Raman spectroscopy was performed with a Horiba microscope using a 632.8 nm HeNe ion laser. PL spectra were measured using a Horiba Jobin Yvon fully automated spectrometer fitted with an InGaAs PMT detector cooled to −30 °C to reduce noise. A 532 nm continuous wave diode pumped solid state (CW-DPSS) laser was used as an excitation source. Capacitance–voltage parameters were evaluated using an Agilent E4980a LCR meter operating at 100 kHz with a bias range from 0.5 to −1 V. External quantum efficiency measurements were performed using a Bentham PVE300 spectral response system (calibrated using a Si–InGaAs reference cell).

### Electron Microscopy

TEM lamellae prior to the *in situ* TEM observation were mounted on MEMS chips according to the procedure described elsewhere^35,36^ using a Zeiss Crossbeam 540 FIB-SEM. The devices were initially protected with electron and ion beam deposited Pt films inside the FIB, followed by milling from a coarse current of 15 nA at 30 kV to a final low-kV polishing at 5 kV and 10 pA, with decreasing current and voltage during the milling process. The HAADF-STEM images were recorded in a JEOL ARM300 equipped of a probe and image correctors. The STEM–EDX signal was recorded using a JEOL-EDX detector installed on the JEOL ARM300 with an 90 mrad semiangle inner collection angle. The *in situ* annealing experiments were performed using a dedicated MEMS-based TEM holder that allowed the temperature to be controlled and the specimen to be biased (not used in this study). More details about the TEM holder and the annealing procedure are described elsewhere.^37^

## Supporting Information Available

Evolution of solar cell parameters with increasing annealing temperature; log *J*–*V* plots of a new, degraded, and annealed CZTSSe device fitted with the two-diode model; HAADF-STEM cross section images of CZTSSe lamellae at RT and 225 °C; peak fitting of the Raman spectra with a box plot of the B mode Raman peak height; peak fitting of PL spectra; bandgap value extraction of CZTSSe absorbers in the degraded and annealed solar cells; device simulation parameters (PDF)
