# Supplementary material for: Recovery Mechanisms in Aged Kesterite Solar Cells
Source: ACS Appl Energy Mater. 2022 Mar 8;5(5):5404–14. doi: 10.1021/acsaem.1c03247 (PMC9131304; doi:10.1021/acsaem.1c03247)
Supplement: Supplementary file 1 — ae1c03247_si_001.pdf [file ae1c03247_si_001.pdf]

Recovery mechanisms in aged kesterite solar cells - Supporting Information

Stephen Campbell,[†,¶] Martial Duchamp,[‡] Bethan Ford,[†] Michael Jones,[†] Linh Lan Nguyen,[‡] Matthew C. Naylor,[†] Xinya Xu,[†] Pietro Maiello,[†] Guillaume Zoppi,[†] Vincent Barrioz,[†] Neil S. Beattie,[*,†] and Yongtao Qu[*,†,¶]

†*Department of Mathematics, Physics and Electrical Engineering, Northumbria University, Newcastle upon Tyne NE1 8ST, United Kingdom*

‡*Laboratory for In Situ and Operando Electron Nanoscopy, School of Materials Science and Engineering, Nanyang Technological University, 637371, Singapore*

¶*These authors contributed equally*

E-mail: neil.beattie@northumbria.ac.uk; y.qu@northumbria.ac.uk

Supporting Information

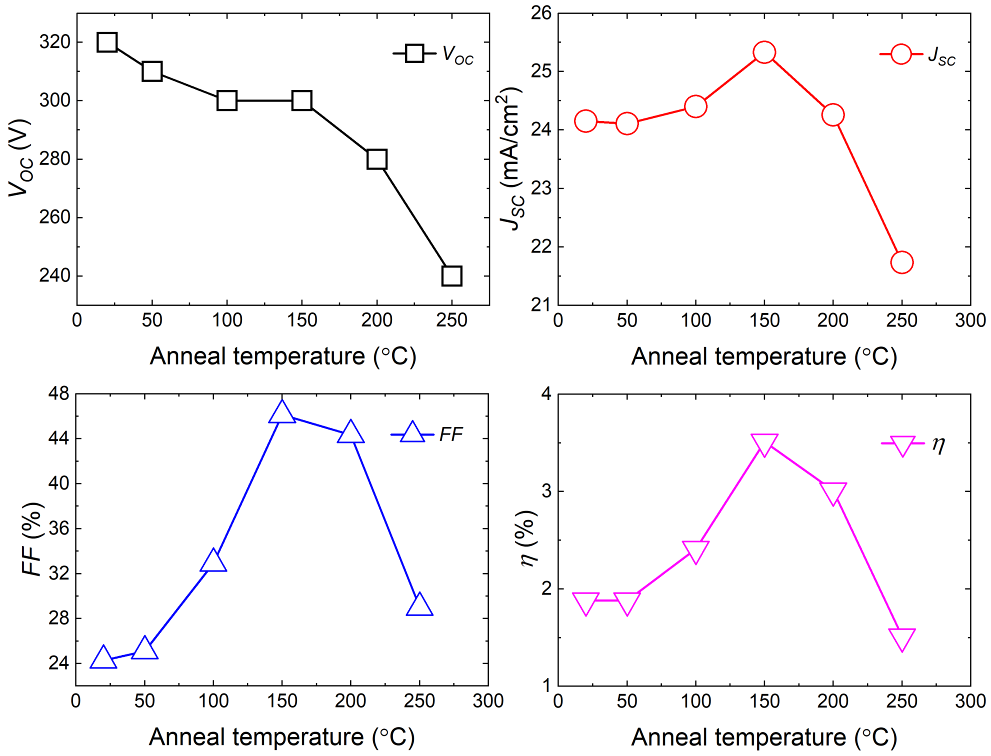

Figure S1: Evolution of solar cell parameters with increasing annealing temperature for a typical degraded CZTSSe solar cell.

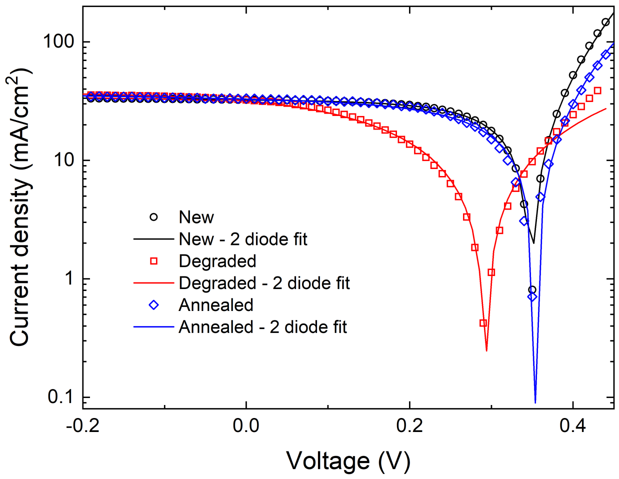

Figure S2: Log J-V plots of a new, degraded and annealed CZTSSe device fitted with the two diode model Equation 3 to extract J-V parameters J_{01} and J_{02}.

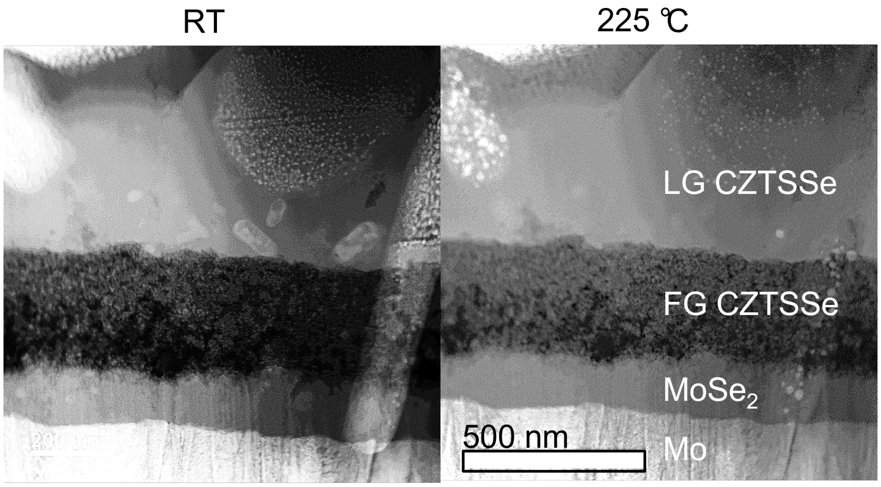

Figure S3: HAADF-STEM images of CZTSSe solar cell lamellae annealed *in-situ* in a TEM recorded at room temperature and 225 °C.

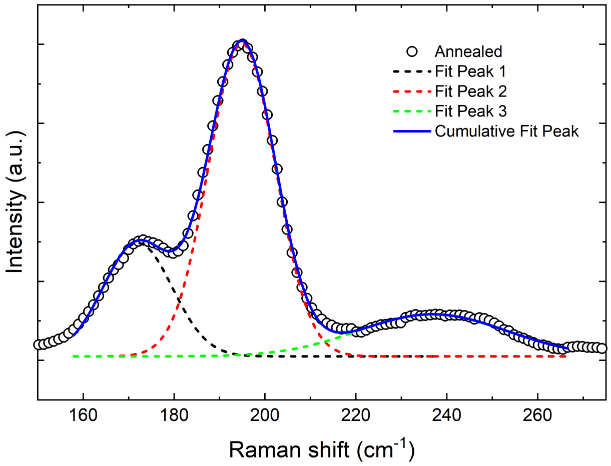

(a)

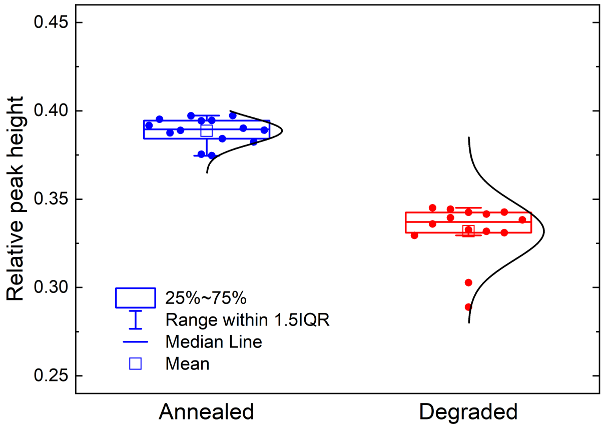

(b)

Figure S4: (a) Example Gaussian peak fitting of the Raman spectra for an annealed CZTSSe solar cell and (b) box plot of relative height of B mode peak ∼172 cm^{-1} in Raman spectra for multiple measurements of degraded and annealed CZTSSe devices.

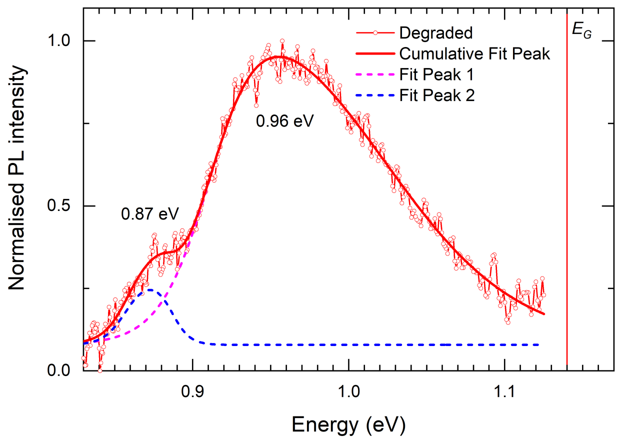

Figure S5: An example room temperature PL spectrum of the degraded solar cell showing the bandgap E_G of the associated CZTSSe absorber. PL spectra were fitted using two asymmetric double sigmoidal functions. The presence of a lower energy peak at 0.87 eV (not observed in the annealed sample) indicates the presence of an additional Cu-related defect in the degraded absorber.[1]

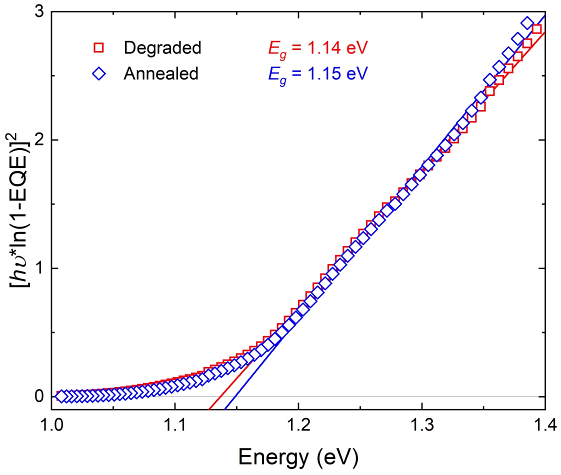

Figure S6: Plots of $[h\upsilon^{*}ln(1-EQE)]^2$ versus $h\upsilon$ to determine E_G values of CZTSSe absorbers in the degraded and annealed solar cell.

Table S1: Device simulation parameters, d: layer thickness, E_g: bandgap, χ: electron affinity, $\varepsilon/\varepsilon_0$: dielectric constant, $m_e{}^*/m_0$: effective mass, μ: carrier mobility, $N_{A/D}$: apparent doping density D:donor A:Acceptor, N_t: bulk defect density, Φ_H: back contact barrier height, E_t: defect energy level relative to CB/VB, σ: capture cross section and N_{int}: interface defect concentration. Subscripts e and h are electron and hole, respectively.

Layer properties	CZTSSe-D	CZTSSe-A	CdS	i-ZnO	ITO
d (μm)	1.200	1.200	0.070	0.035	0.200
E_g (eV)	1.13[a]	1.14[a]	2.42[a]	3.37[b]	3.72[b]
χ (eV)	4.6[c]	4.6[c]	4.5[c]	4.7[d]	4.5[d]
$\varepsilon/\varepsilon_0$	8.5[e]	8.5[e]	9.0[b]	9.0[d]	9.4[d]
$m_e{}^*/m_0$	0.1[e]	0.1[e]	0.25[b]	0.275[d]	0.22[d]
$m_h{}^*/m_0$	0.32[e]	0.32[e]	0.7[b]	0.59[d]	0.58[d]
μ_e (cm^2/Vs)	80[b]	80[b]	160[b]	200[b]	30[b]
μ_h (cm^2/Vs)	25[b]	25[b]	15[b]	93[b]	5[b]
$N_{A/D}$ (cm^{-3})	A: 7.2 x10^{15}[f]	A: 1.1 x10^{16}[f]	D: 1 x10^{17}[b]	D: 1 x10^{18}[b]	D: 1 x10^{21}[b]
Φ_H (meV)	109	44			
Bulk defects (single level)					
N_t (cm^{-3})	A: 10^{14}[g]	A: 10^{14}[g]	D: 10^{17}[b]	D: 10^{17}[b]	D: 10^{16}[b]
E_t (cm^{-3})	0.09[g]	0.09[g]	0.10[b]	0.10[b]	0.10[b]
N_t (cm^{-3})	A: 10^{14}[g]	A: 10^{14}[g]			
E_t (cm^{-3})	0.18[g]	0.18[g]			
N_t (cm^{-3})	D: 10^{15}[b]	D: 10^{15}[b]			
E_t (cm^{-3})	0.63[b]	0.63[b]			
Bulk defect (distribution:erfc 200 nm from CdS/CZTSSe interface) Cd_{Zn}					
N_t (cm^{-3})	A: varied	A: varied			
E_t (cm^{-3})	0.43[b]	0.43[b]			
σ_e (cm^2)	10^{-13}[b]	10^{-13}[b]	10^{-17}[b]	10^{-15}[b]	10^{-17}[b]
σ_h (cm^2)	10^{-14}[b]	10^{-14}[b]	10^{-11}[b]	10^{-12}[b]	10^{-12}[b]
Interface defects between CZTSSe and CdS (Gaussian distribution throughout interface)					
N_{int} (cm^{-3})	D: varied	D: varied	D: varied		
σ_e (cm^2)	10^{-13}	10^{-13}	10^{-13}		
σ_h (cm^2)	10^{-15}	10^{-15}	10^{-15}		

[a]Experimentally determined from UV-VIS and EQE measurements
[b]Reference[2]
[c]Reference[3]
[d]Reference[4]
[e]Reference[5]
[f]Experimentally determined from C-V measurements
[g]Experimentally determined from PL measurements and DLTS in reference[6]

References

(1) Chen, S.; Walsh, A.; Gong, X.-G.; Wei, S.-H. Classification of Lattice Defects in the Kesterite Cu_2ZnSnS_4 and $Cu_2ZnSnSe_4$ Earth-Abundant Solar Cell Absorbers. *Advanced Materials* **2013**, *25*, 1522–1539.

(2) Kanevce, A.; Repins, I.; Wei, S.-H. Impact of bulk properties and local secondary phases on the $Cu_2(Zn,Sn)Se_4$ solar cells open-circuit voltage. *Solar Energy Materials and Solar Cells* **2015**, *133*, 119–125.

(3) Erkan, M. E.; Chawla, V.; Scarpulla, M. A. Reduced defect density at the CZTSSe/CdS interface by atomic layer deposition of Al_2O_3. *Journal of Applied Physics* **2016**, *119*, 194504.

(4) Hossain, M.; Chelvanathan, P.; Zaman, M.; Karim, M.; Alghoul, M.; Amin, N. Prospects of indium sulphide as an alternative to cadmium sulphide buffer layer in CIS based solar cells from numerical analysis. *Chalcogenide Letters* **2011**, *8*, 315–324.

(5) Persson, C. Electronic and optical properties of Cu_2ZnSnS_4 and $Cu_2ZnSnSe_4$. *Journal of Applied Physics* **2010**, *107*, 053710.

(6) Campbell, S.; Qu, Y.; Major, J. D.; Lagarde, D.; Labbé, C.; Maiello, P.; Barrioz, V.; Beattie, N. S.; Zoppi, G. Direct evidence of causality between chemical purity and band-edge potential fluctuations in nanoparticle ink-based $Cu_2ZnSn(S,Se)_4$ solar cells. *Journal of Physics D: Applied Physics* **2019**, *52*, 135102.
